# Placental DNA methylation signatures of maternal smoking during pregnancy and potential impacts on fetal growth

**DOI:** 10.1038/s41467-021-24558-y

**Published:** 2021-08-24

**Authors:** Todd M. Everson, Marta Vives-Usano, Emie Seyve, Andres Cardenas, Marina Lacasaña, Jeffrey M. Craig, Corina Lesseur, Emily R. Baker, Nora Fernandez-Jimenez, Barbara Heude, Patrice Perron, Beatriz Gónzalez-Alzaga, Jane Halliday, Maya A. Deyssenroth, Margaret R. Karagas, Carmen Íñiguez, Luigi Bouchard, Pedro Carmona-Sáez, Yuk J. Loke, Ke Hao, Thalia Belmonte, Marie A. Charles, Jordi Martorell-Marugán, Evelyne Muggli, Jia Chen, Mariana F. Fernández, Jorg Tost, Antonio Gómez-Martín, Stephanie J. London, Jordi Sunyer, Carmen J. Marsit, Johanna Lepeule, Marie-France Hivert, Mariona Bustamante

**Affiliations:** 1 Gangarosa Department of Environmental Health, Rollins School of Public Health at Emory University, Atlanta, GA USA; 2 Center for Genomic Regulation (CRG), Barcelona Institute of Science and Technology, Barcelona, Spain; 3 Universitat Pompeu Fabra, Barcelona, Spain; 4 CIBER Epidemiología y Salud Pública (CIBERESP), Madrid, Spain; 5 University Grenoble Alpes, Inserm, CNRS, IAB, Grenoble, France; 6 Department of Population Medicine, Harvard Medical School, Harvard Pilgrim Health Care Institute, Boston, MA USA; 7 Division of Environmental Health Sciences, School of Public Health, University of California, Berkeley, Berkeley, CA USA; 8 Andalusian School of Public Health, Granada, Spain; 9 Instituto de Investigación Biosantaria (ibs.GRANADA), Granada, Spain; 10 Epigenetics Group, Murdoch Children’s Research Institute, Parkville, VIC Australia; 11 Department of Paediatrics, University of Melbourne, Parkville, VIC Australia; 12 IMPACT – the Institute for Mental and Physical Health and Clinical Translation, Deakin University, Geelong, VIC Australia; 13 Department of Environmental Medicine and Public Health, Icahn School of Medicine at Mount Sinai, New York, NY USA; 14 Department of Obstetrics & Gynecology, Geisel School of Medicine at Dartmouth College, Lebanon, NH USA; 15 University of the Basque Country (UPV/EHU), Leioa, Spain; 16 Biocruces-Bizkaia Health Research Institute, Barakaldo, Spain; 17 Public Health Division of Gipuzkoa, Basque Government, San Sebastian, Spain; 18 Université de Paris, CRESS, INSERM, INRAE, Paris, France; 19 Department of Medicine, University of Sherbrooke, Sherbrooke, QC Canada; 20 Reproductive Epidemiology, Murdoch Children’s Research Institute, Parkville, VIC Australia; 21 Department of Epidemiology, Geisel School of Medicine at Dartmouth College, Hanover, NH USA; 22 Department of Statistics and Computational Research, Universitat de València, València, Spain; 23 Epidemiology and Environmental Health Joint Research Unit, FISABIO-Universitat Jaume I-Universitat de València, València, Spain; 24 Department of Biochemistry and Functional Genomics, University of Sherbrooke, Sherbrooke, QC Canada; 25 Bioinformatics Unit, GENYO. Centre for Genomics and Oncological Research, Pfizer, University of Granada, Andalusian Regional Government, Granada, Spain; 26 Department of Statistics, Faculty of Sciences, University of Granada, Granada, Spain; 27 Department of Genetics and Genomic Sciences, Icahn School of Medicine at Mount Sinai, New York, NY USA; 28 IUOPA University of Oviedo, Oviedo, Spain; 29 Atrys Health S.A., Barcelona, Spain; 30 Biomedical Research Centre (CIBM) and School of Medicine, University of Granada, Granada, Spain; 31 Laboratory for Epigenetics and Environment, Centre National de Recherche en Génomique Humaine, CEA – Institut de Biologie François Jacob, Evry, France; 32 Genomics Unit, GENYO. Centre for Genomics and Oncological Research, Pfizer, University of Granada, Andalusian Regional Government, Granada, Spain; 33 Division of Intramural Research, National Institute of Environmental Health Sciences, National Institutes of Health, Department of Health and Human Services, Durham, NC USA; 34 ISGlobal, Barcelona Institute for Global Health, Barcelona, Spain; 35 Hospital del Mar Medical Research Institute (IMIM), Barcelona, Spain; 36 Department of Epidemiology, Rollins School of Public health at Emory University, Atlanta, GA USA; 37 Diabetes Unit, Massachusetts General Hospital, Boston, MA USA

**Keywords:** Epigenetics, Epigenomics, Risk factors

## Abstract

Maternal smoking during pregnancy (MSDP) contributes to poor birth outcomes, in part through disrupted placental functions, which may be reflected in the placental epigenome. Here we present a meta-analysis of the associations between MSDP and placental DNA methylation (DNAm) and between DNAm and birth outcomes within the Pregnancy And Childhood Epigenetics (PACE) consortium (*N* = 1700, 344 with MSDP). We identify 443 CpGs that are associated with MSDP, of which 142 associated with birth outcomes, 40 associated with gene expression, and 13 CpGs are associated with all three. Only two CpGs have consistent associations from a prior meta-analysis of cord blood DNAm, demonstrating substantial tissue-specific responses to MSDP. The placental MSDP-associated CpGs are enriched for environmental response genes, growth-factor signaling, and inflammation, which play important roles in placental function. We demonstrate links between placental DNAm, MSDP and poor birth outcomes, which may better inform the mechanisms through which MSDP impacts placental function and fetal growth.

## Abstract (web summary)

Maternal smoking during pregnancy contributes to poor birth outcomes. Here the authors perform a meta-analysis of the associations between maternal smoking during pregnancy and placental DNA methylation and identify links between these and poor birth outcomes, which may better inform the mechanisms through which smoking impacts placental function and fetal growth.

## Introduction

Almost one in ten mothers smoke during pregnancy, with state-specific prevalence ranging from as low as 1.8% to as high as 27.1% in the USA^1^, while in Europe, the prevalence of maternal smoking during pregnancy (MSDP) ranges between 4.2 and 18.9% (ref. ^2^). Consequently, the numerous health effects of MSDP convey a significant public health concern. The impact of this exposure on fetal development has been the source of significant investigation, resulting in MSDP being recognized as a cause of multiple negative pregnancy and birth outcomes^3^.

The mechanisms that underlie this reproductive and developmental toxicity are partially understood, and include molecular and anatomical changes of the placenta^4,5^. In addition, experimental mouse models have recently highlighted the critical roles of proper placental function in ensuring successful pregnancy outcomes^6^. Epigenetic responses to prenatal exposures have emerged as potential intermediate links between early life exposures and developmental health outcomes, and epidemiologic studies of DNAm, particularly multi-cohort collaborative efforts, are powerful approaches to investigate these types of research questions^7^. Most studies of MSDP and epigenetics have focused on DNA methylation (DNAm) in cord blood, although some studies of placenta, peripheral blood, and lung tissues have also been performed^8^. The Pregnancy and Childhood Epigenetics (PACE) consortium^9^ published a large meta-analysis identifying thousands of MSDP-associated variations in DNAm within cord blood and child peripheral blood^10^. However, the placental epigenome has not been as thoroughly studied, although the placenta is likely a critical target organ of MSDP-associated toxicity. A handful of prior studies have examined the relationships between MSDP and (DNAm) in human placenta^11–14^, identifying MSDP-associated CpGs some of which have been suggested to partially mediate the effects of MSDP on lower birth weight (BW)^15^.

These studies have begun to characterize the impact that MSDP has on the human placental epigenome, but have been limited by small sample sizes. We aimed to address this gap by performing a fixed effects meta-analysis examining the relationships between MSDP and variations in the placental methylome across seven independent studies that are members of the PACE consortium. We also aimed to gain insights into the potential biological processes that might be affected and the relationships with birth outcomes by performing additional analyses with nearby mRNA expression, as well as functional, regulatory, and phenotypic enrichment, and a secondary meta-analysis of the associations between placental DNAm and birth outcomes. Here, we demonstrate that placental DNAm is associated with MSDP throughout the genome, and these differences in DNAm are associated with gestational age (GA) and birth size metrics. We also show that many of these CpGs are correlated with nearby gene expression and are enriched for genes involved in environmental response, growth factor signaling, and inflammation. Further studies are needed to assess potential causality and mediation.

## Results

### Study population

Seven American, Australian, and European studies (*N* = 1700) contributed to the epigenome-wide association study (EWAS) linking MSDP to placental DNAm: including Asking Questions about Alcohol in pregnancy (AQUA)^16^, Study on the prenatal and early postnatal determinants of child health and development (EDEN)^17^, Genetics of Glucose regulation in Gestation and Growth (Gen3G)^18^, Genetics, Early Life Environmental Exposures, and Infant Development in Andalusia (GENEIDA), Environment and Childhood Project (INMA)^19^, New Hampshire Birth Cohort Study (NHBCS)^20^, and Rhode Island Child Health Study (RICHS)^21^. For this meta-analysis, 344 (20.2%) mothers reported any MSDP, defined as any cigarette smoking during any trimester of pregnancy. Any MSDP tended to be less prevalent in the cohorts from Canada and the USA compared to those from Australia and Europe (Table 1). Three cohorts (*N* = 795, EDEN, GENEIDA, and INMA) contributed to the EWAS of sustained MSDP, defined as maternal smoking throughout pregnancy, among which 163 (20.5%) mothers reported sustained MSDP. Distributions of covariates by cohort are provided in the Supplementary Materials (Supplementary Data 1). The comparison group for models of any or sustained MSDP included all mothers that did not report smoking cigarettes during any trimester.

**Table 1:** Frequencies of any and sustained MSDP within participating cohorts.

Cohort	Country	Any MSDP			Sustained MSDP		
		Total *N*	*N* (%) nonsmokers	*N* (%) smokers	Total *N*	*N* (%) nonsmokers	*N* (%) smokers
AQUA	Australia	99	75 (75.76%)	24 (24.24%)	—	—	—
EDEN	France	647	446 (68.93%)	201 (31.07%)	570	446 (68.93%)	124 (21.75%)
Gen3G	Canada	151	138 (91.39%)	13 (8.61%)	—	—	—
GENEIDA	Spain	87	67 (77.01%)	20 (22.99%)	82	67 (77.01%)	15 (18.29%)
INMA	Spain	166	119 (71.69%)	47 (28.31%)	143	119 (71.69%)	24 (16.78%)
NHBCS	USA	310	290 (93.55%)	20 (6.45%)	—	—	—
RICHS	USA	240	221 (92.08%)	19 (7.92%)	—	—	—
TOTAL		1700	1356 (79.76%)	344 (20.24%)	795	632 (79.50%)	163 (20.50%)

*MSDP* maternal smoking during pregnancy.

### Genome-wide DNAm meta-analyses

**Fig. 1:**
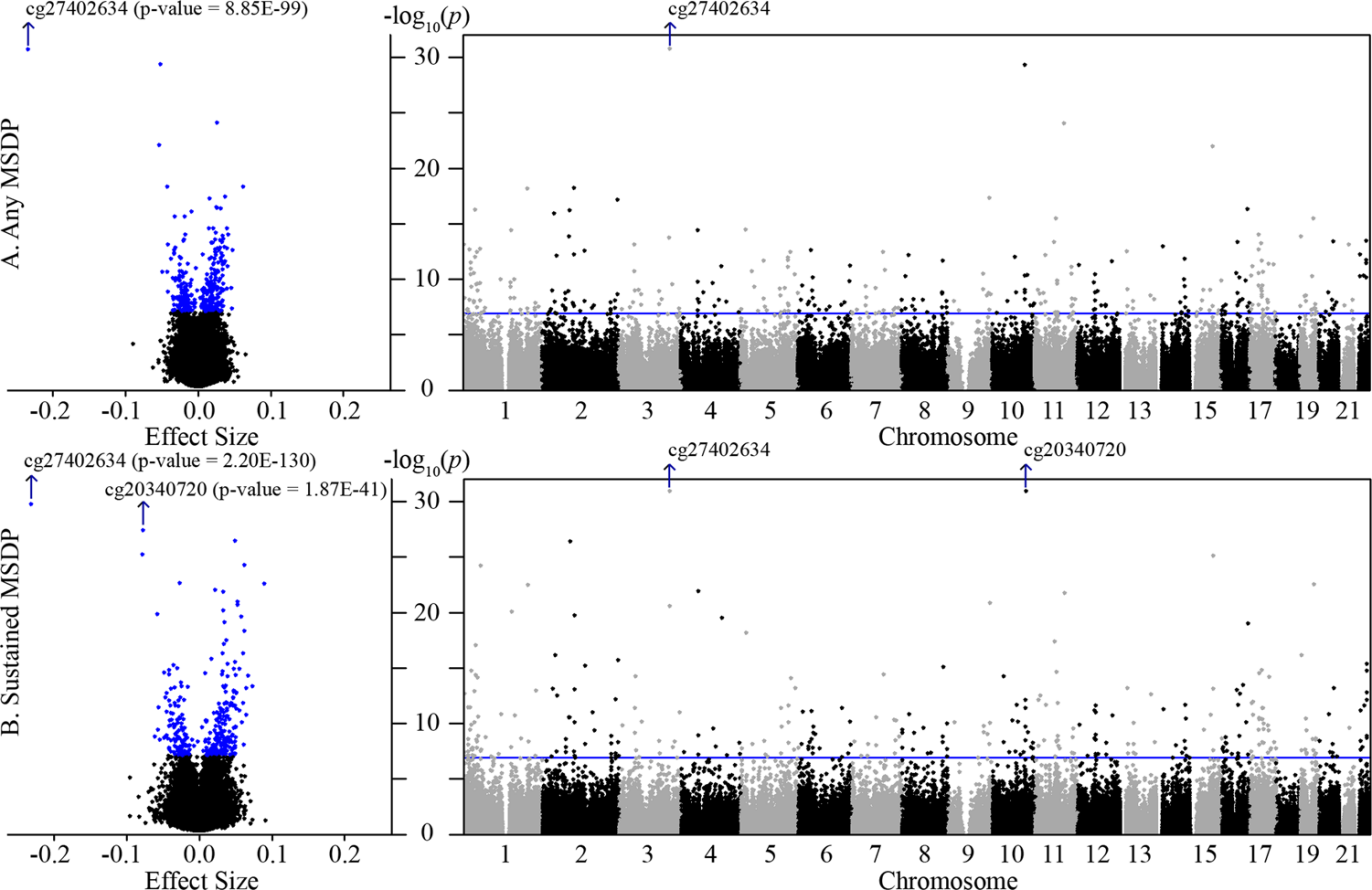
Volcano and Manhattan plots of the inverse-variance fixed meta-analysis results for any and sustained MSDP. **A** Placental DNAm associations with any MSDP (total *N* = 1700 independent samples from seven independent studies; exposed = 344). **B** Placental DNAm associations with sustained MSDP (total *N* = 795 independent samples from three independent studies; exposed = 163). For both analyses, models were adjusted for maternal age, parity, maternal education, putative cellular heterogeneity, and residual bias. In the volcano plots, the *x*-axes show the estimated mean difference in DNAm (effect size), when comparing mothers that smoked during pregnancy (MSDP) to those that did not, with a possible range between 0 and 1, while the *x*-axes in the Manhattan plots represent genomic location; both plots share the same *y*-axes with −log_10_(*p* values). Bonferroni thresholds for statistical significance are shown as blue dots and a blue horizontal line, for volcano and Manhattan plots, respectively. The *y*-axes were truncated to a minimum *p* value of 1 × 10^−30^ (or maximum −log_10_(*p*) of 30), to allow for better visualization of the majority of our results.

We produced four statistical models for each CpG site, regressing DNAm on both any and sustained MSDP, with and without adjustment for putative cellular heterogeneity, which was estimated with a reference-free deconvolution algorithm, RefFreeCellMix^22^. All models were adjusted for maternal age, parity, and maternal education. Genomic inflation factors from the cohort-specific models (ranging from *λ* = 0.824 to 2.478) and meta-analyses (ranging from *λ* = 2.002 to 2.839; Supplementary Data 2 and Supplementary Fig. 1) revealed potential residual confounding and inflation of test statistics. All EWAS results with Bonferroni-corrected *p* values < 0.05 are included in the Supplementary Materials (Supplementary Data 3–6). Heterogeneity in the associations across cohorts was lowest for the models that were adjusted for RefFreeCellMix (Supplementary Data 7), and thus we utilized the results from these adjustment models for all downstream analyses. To correct for residual bias and inflation, we, then, implemented BACON, which estimates an empirical null distribution to the data^23^. This substantially reduced inflation among cohort-specific results (ranged 0.957–1.228), and meta-analyses of these BACON-corrected estimates yielded 443 CpGs that were associated with any or sustained MSDP after Bonferroni correction (Supplementary Data 8 and Fig. 1). While 443 CpGs is a large number of sites to identify in an EWAS, this is similar in size to the 568 CpGs identified in a previous PACE meta-analysis of cord blood DNAm associated with MSDP^10^. Sustained MSDP yielded similar, but stronger effects for 93% of these 443 CpGs (Supplementary Fig. 2).

We did not adjust for GA because we hypothesized that it was likely downstream of the epigenetic response to MSDP or possibly on the causal pathway between MSDP and changes in DNAm; in either case, it would likely result in overadjustment^24^. However, we did explore whether additional adjustment for GA altered the observed relationships in four of our cohorts (EDEN, INMA, NHBCS, and RICHS). We found that these additional adjustments had almost no effect on the estimates of differential DNAm, with no apparent attenuation of effects toward the null (Supplementary Fig. 3). Thus, MSDP-associated differences in GA could not explain the observed relationships between DNAm and MSDP.

**Fig. 2:**
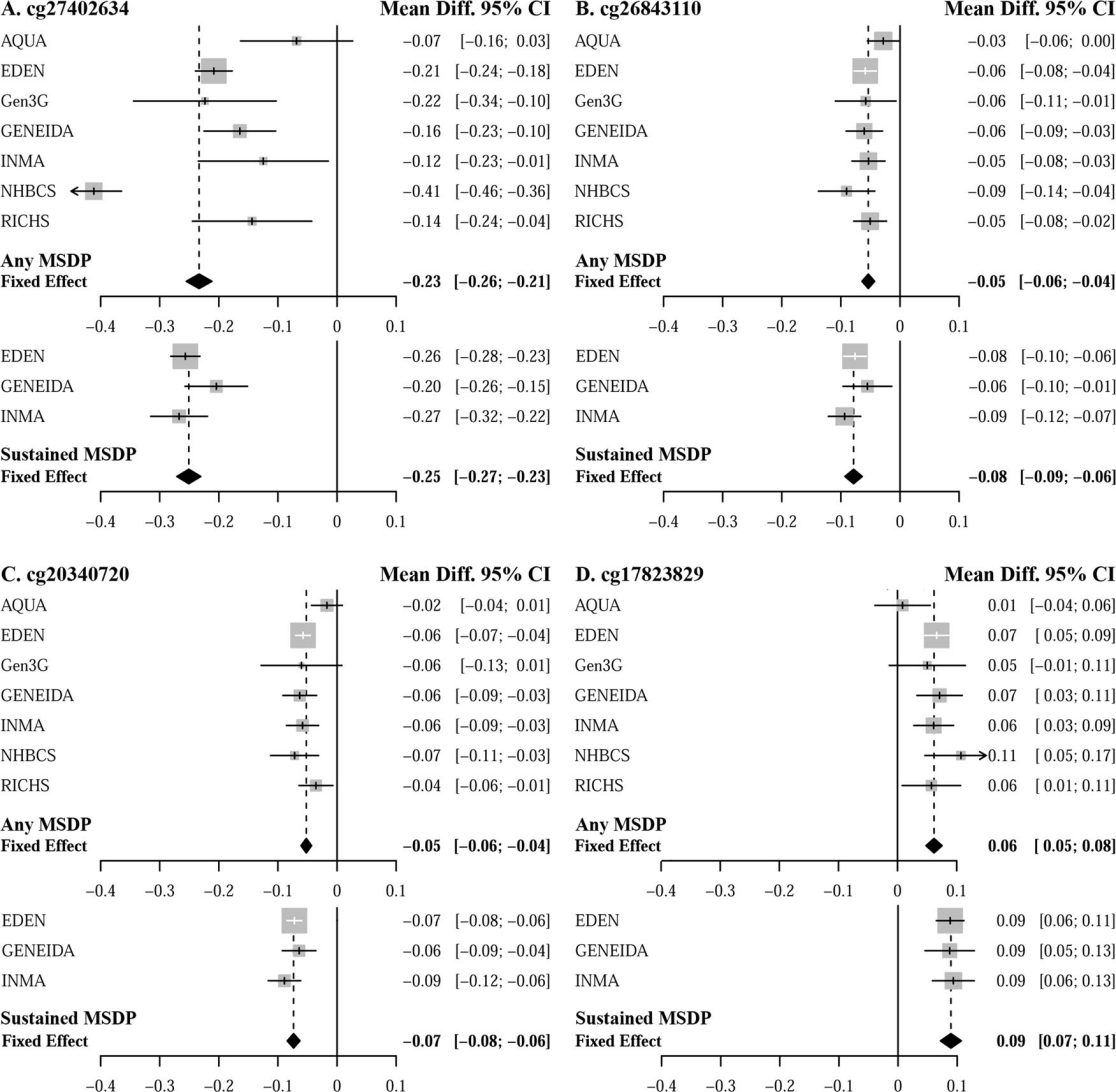
Forest plots of cohort specific and inverse-variance fixed-effect meta-analysis estimates of associations between MSDP with placental DNAm. Estimated differential DNAm (Mean Diff.) and 95% confidence intervals (95% CI) at **A** cg27402634, **B** cg26843110, **C** cg20340720, and **D** cg17823829 with any MSDP and sustained MSDP. All models were adjusted for maternal age, parity, maternal education, putative cellular heterogeneity, and for residual bias. The mean difference represents the estimated difference in the proportion of DNAm at each CpG when comparing mothers that smoked during pregnancy (MSDP), to those that did not smoke during pregnancy via linear regression.

The most notable association was observed at cg27402634, located upstream of the *LEKR1* gene and the noncoding RNA *LINC00886*, which showed the largest differential DNAm and smallest *p* values in all meta-analyses. Placentas that were exposed to any MSDP had 23.33% lower DNAm (95% CI: 21.17–25.51% lower DNAm; inverse-variance fixed-effect meta-analysis *p* value = 8.85E−99) and those exposed to sustained MSDP had 25.08% lower DNAm (95% CI: 23.06–27.11% lower DNAm; inverse-variance fixed-effect meta-analysis *p* value = 2.20−E130), when compared to mothers that did not smoke at all during pregnancy. Although all cohorts observed substantial hypomethylation with MSDP at this CpG, the actual estimates of the associations were highly variable between cohorts for models of any MSDP (Cochran’s *Q* test *p* value = 1.74E−15), but relatively consistent for models of sustained MSDP (Cochran’s *Q* test *p* value = 1.61E−01; Fig. 2A). Overall, we observed consistency in the associations across cohorts for the vast majority of the 443 CpGs: 93% and 96% of the 443 CpGs yielded heterogeneity *p* values > 0.01, for any and sustained MSDP, respectively. In addition to cg27402634, we highlight those relationships that yielded that largest magnitudes of association: |*β*_Any MSDP_| > 0.05 for cg26843110 (*EDC3*), cg20340720 (*WBP1L*), and cg17823829 (*KDM5B*; Fig. 2B–D). We identified numerous other noteworthy relationships but due to the large number of genome-wide significant associations, we highlight the relationship among the 20 most statistically significant CpGs from the primary meta-analysis of any MSDP going forward (Table 2), while results for all BACON-adjusted genome-wide significant CpGs, along with detailed annotations, are included in the Supplementary Materials (Supplementary Data 8).

**Table 2:** Top 20 meta-analysis results from models of any and sustained MSDP.

Annotations			Any MSDP		Sustained MSDP		% Diff.
CpG ID	Location	Gene (region)	*β*_1_	*p*	*β*_1_	*p*	
cg25112191	chr1:151804260	*RORC* (1st exon; 5′UTR)	0.021	3.56E−15	0.033	7.22E−21	55.1
cg17823829	chr1:202765754	*KDM5B* (body)	0.062	6.29E−19	0.089	2.74E−23	44.8
cg26045080	chr1:36807363	*STK40* (body)	0.026	5.26E−17	0.034	8.48E−18	30.4
cg00534380	chr2:101766586	*TBC1D8* (body)	−0.043	5.85E−19	−0.058	1.66E−20	35.7
cg19246018	chr2:240031588	*HDAC4* (body)	0.016	6.93E−18	0.017	1.86E−16	5.6
cg06202585	chr2:38325802	2p22.2	−0.009	1.08E−16	−0.009	1.78E−06	0.1
cg23752985	chr2:85803571	*VAMP8* (TSS1500)	−0.018	1.30E−14	−0.023	2.51E−11	28.7
cg00666842	chr2:88366145	*SMYD1* (TSS1500)	0.031	5.84E−17	0.049	3.35E−27	57.2
cg27402634	chr3:156536860	3q25.31	−0.233	8.85E−99	−0.251	2.20E−130	7.5
cg09491670	chr4:53529646	4q12	0.015	3.71E−15	0.022	1.06E−22	45.1
cg25585967	chr5:14452105	*TRIO* (body)	0.040	3.50E−15	0.062	5.97E−19	54.8
cg14214914	chr9:131870304	*CRAT* (body)	0.037	4.84E−18	0.052	1.21E−21	39.1
cg20340720	chr10:104512523	*WBP1L* (body)	−0.052	4.44E−30	−0.074	1.87E−41	41.3
cg26648103	chr11:66791718	*SYT12* (5′UTR)	−0.033	3.29E−16	−0.042	2.06E−15	28.4
cg26115089	chr11:93846406	*HEPHL1* (3′UTR)	0.026	9.16E−25	0.033	1.53E−22	23.9
cg26843110	chr15:74935742	*EDC3* (body)	−0.054	1.05E−22	−0.079	6.56E−26	46.3
cg26433445	chr16:81764289	16q23.3	0.025	4.72E−17	0.035	8.84E−20	39.9
cg24177452	chr17:27494295	*MYO18A* (5′UTR)	0.024	9.42E−15	0.032	2.55E−15	30.1
cg22018329	chr19:3116716	*GNA11* (body)	0.041	1.41E−14	0.060	6.48E−17	45.6
cg03313447	chr19:41829042	*CCDC97* (3′UTR)	−0.019	3.28E−16	−0.027	2.70E−23	43.0

All models were adjusted for maternal age, parity, education, putative cellular heterogeneity, and residual bias; CpGs that were not annotated with a gene name in the Illumina 450 K annotation file have been annotated with their genomic region (i.e., 2p22.2).

*β*_*1*_ coefficient of the association between DNAm and MSDP, which represent the estimated difference in the proportion of DNAm at each CpG when comparing mothers that smoked during pregnancy (MSDP), to those that did not smoke during pregnancy; *p* values from inverse-variance fixed-effect meta-analyses, *% Diff.* the percent difference in effect size when comparing the *β*_1_ for sustained MSDP to the *β*_1_ for any MSDP (% Diff.), *MSDP* maternal smoking during pregnancy.

### Expression quantitative trait methylation (eQTM) analyses

We then performed expression quantitative trait methylation (eQTM) analyses, testing whether the DNAm levels at MSDP-associated CpGs were associated with the expression of nearby mRNA (within 250 kb of the CpG) from 194 placental samples in the RICHS cohort. We mapped each CpG to the gene that was most strongly associated with DNAm levels if the association produced a *p* value < 0.05, then used a Bonferroni-corrected threshold to identify those eQTMs with the strongest evidence of a relationship. Among the 421 CpGs that were within 250 kb of a transcription start site (TSS), 258 CpGs were mapped to eQTM genes (*p* values < 0.05), 40 of which were significant after Bonferroni correction (*α* = 1.43E−05; Supplementary Data 9). The majority of mapped eQTMs exhibited inverse associations (65%) and statistical significance was strongest for CpGs that were closest to the TSS (Supplementary Fig. 4). Among the top 20 CpGs from our meta-analysis, 15 mapped to eQTM genes (Table 3) and 5 of which were significant at the Bonferroni-adjusted threshold: *SH3D21*, *TBC1D8*, *USP46*, *CRAT*, and *TGFB1*.

**Table 3:** Results from eQTM models, DNAm versus GA at birth, and DNAm versus BW.

CpG ID	eQTM gene	Ann. gene	eQTM *β*_1_	eQTM *p*	GA *β*_1_	GA *p*	BW *β*_1_	BW *p*
cg25112191	*RORC*	*RORC*	−7.28	5.17E−05	0.49	4.59E−01	−2.70	**4.08E−05**
cg17823829	*CYB5R1*	*KDM5B*	0.39	2.99E−02	−1.18	**9.11E−06**	−1.02	**9.72E−05**
cg26045080	*SH3D21*	*STK40*	−2.43	**2.42E−07**	−1.41	4.19E−03	−0.93	5.70E−02
cg00534380	*TBC1D8*	*TBC1D8*	−2.46	**1.22E−08**	3.53	**9.26E−20**	1.48	1.62E−04
cg19246018	NA	*HDAC4*	1.53	4.05E−01	−1.39	1.74E−02	−1.03	7.67E−02
cg06202585	*CYP1B1*	2p22.2	−12.78	3.09E−03	2.23	5.94E−02	1.53	1.97E−01
cg23752985	*CAPG*	*VAMP8*	4.23	3.24E−03	5.47	**1.40E−15**	−0.31	6.62E−01
cg00666842	NA	*SMYD1*	−1.27	6.78E−02	0.45	2.94E−01	−0.44	3.07E−01
cg27402634	*LEKR1*	3q25.31	−2.34	3.41E−04	−0.28	1.41E−01	0.92	**6.71E−07**
cg09491670	*USP46*	4q12	−3.72	**2.55E−06**	−0.88	2.22E−01	−0.65	3.64E−01
cg25585967	NA	*TRIO*	0.95	1.54E−01	−0.97	8.64E-03	−1.35	2.06E−04
cg14214914	*CRAT*	*CRAT*	−3.20	**1.46E−10**	−0.96	1.35E−02	−0.23	5.49E−01
cg20340720	*WBP1L*	*C10orf26*	−0.69	2.81E−03	0.49	1.88E−01	1.86	**2.42E−07**
cg26648103	*SYT12*	*SYT12*	−1.80	7.31E−03	1.06	1.29E−02	1.66	**7.56E−05**
cg26115089	NA	*HEPHL1*	−0.74	5.20E−01	−1.10	4.43E−02	−1.48	6.71E−03
cg26843110	*UBL7-AS1*	*EDC3*	−1.08	1.18E−02	2.30	**5.09E−12**	1.28	1.19E−04
cg26433445	NA	16q23.3	−0.51	4.40E−01	−0.38	5.45E−01	−2.99	**9.06E−07**
cg24177452	*MYO18A*	*MYO18A*	−0.66	2.36E−02	−0.76	1.24E−01	−0.74	1.29E−01
cg22018329	*AES*	*GNA11*	−0.71	3.78E−03	−1.67	**6.86E−07**	−0.49	1.47E−01
cg03313447	*TGFB1*	*CCDC97*	−9.79	**1.08E−14**	−2.48	7.79E−04	2.29	1.58E−03

We present the regression coefficients and *p* values from the models of eQTMs, gestational age at birth, and birth weight, among the 20 CpGs presented in Table 2. CpGs were mapped to eQTM genes whose expression levels were associated with CpG DNAm levels (eQTM *p* value < 0.05) and to the annotated genes from the Illumina 450 K annotation file; CpGs that were not annotated with a gene name in the Illumina 450 K annotation file have been annotated with their genomic region (i.e., 2p22.2). Associations passing Bonferroni-correction are shown in bold.

*β*_*1*_ regression coefficients from linear models, in which mRNA expression (eQTM), gestational age (GA; inverse normal transformed), and birth weight (BW; *z-*scores) were regressed on DNAm at each CpG, while adjusting for confounders; *p* values (*p*) for eQTM results are from linear regression models, while *p* values (*p*) for GA and BW results are from inverse-variance fixed-effect meta-analysis, *eQTM* expression quantitative trait methylation.

### Functional and regulatory enrichment analyses

Enrichment analyses were performed to gain insights into the biological processes in placenta that may be impacted by MSDP through altered epigenetic regulation. We performed gene-set enrichment analyses using two different gene sets (Supplementary Data 10), first using the genes annotated to the 443 MSDP-associated CpGs according to the Illumina annotation file (284 genes), and second using the genes mapped to these CpGs via the eQTM analyses (211 genes). The first gene list leverages as much of the data as possible that was generated from our most well-powered analysis. Since DNAm is a relatively stable epigenetic feature, this could represent a record of the genes whose methylation levels were perturbed by MSDP across the pregnancy period. On the other hand, gene expression is more dynamic than DNAm and is affected by multiple stimuli. Thus, the second eQTM-mapped gene set likely represents the genes that are influenced by DNAm at these MSDP-associated CpGs at, or close to, the time of birth.

We found that 46 and 9 biological pathways were significantly (*q* value < 0.05) enriched among the Illumina annotated and eQTM-mapped genes, respectively (Supplementary Data 11 and 12). Overall, the eQTM genes were involved in inflammatory activity (aryl hydrocarbon receptor (Ahr) pathway, Th17 cell differentiation, neutrophil degranulation, and platelet degranulation), tyrosine kinase signaling (TYROBP causal network and signaling by receptor tyrosine kinases), carcinogenesis (pathways in clear cell renal cell carcinoma), adipogenesis, and platelets. These pathways included multiple eQTM-mapped genes that were within the Bonferroni-corrected significance threshold; most notably, *TGFB1*, was involved in almost all of these pathways, and *TNFRSF1B* and *ACLY*, were each involved in two of these pathways. The Illumina annotated gene set, which included a larger number of genes annotated to more MSDP-associated CpGs, was additionally enriched with numerous pathways involving growth factor signaling (FGFR, EGF-EGFR, and PDGFR), hormones (aldosterone, insulin, and TSH), immune and inflammatory signaling (IL2 and IL6), MAPK signaling, myometrial and vascular smooth muscle contraction, signal transduction, and cancer pathways. While only one pathway was enriched among both annotated and eQTM genes, signaling by receptor tyrosine kinases (annotated genes *q* value = 0.025 and eQTM-mapped genes *q* value = 0.032, via hypergeometric test), genes from both lists were involved in numerous inflammatory signaling pathways.

To further understand the regulatory landscape for these differentially methylated CpG sites, we used EnrichR to test for enrichment of transcription factor (TF) targets from ENCODE/ChEA databases^25^. The genes annotated to our MSDP-associated CpGs were targets of GATA1 and GATA2, the androgen receptor, TP63, SMAD4, RUNX1, and ZBTB7A (Supplementary Data 13). While the eQTM gene list was not significantly enriched for TF binding, the top TF was RUNX1 (Supplementary Data 14). We then examined whether the MSDP-associated CpG sites were enriched for allele-specific germline differentially methylated regions (gDMR)^26^, regulatory features from the placenta-specific 15-chromatin state annotation from ROADMAP^27^, or placenta-specific partially methylated domains (PMD)^28^, which contain placenta-specific repressed genes (annotated to the results files in Supplementary Data 8). Most notably, the MSDP-associated CpGs were substantially depleted for PMDs (Supplementary Fig. 5), and highly enriched in placental enhancers (Supplementary Fig. 6); both of these are indicators that our set of placental MSDP-associated CpGs occur within highly active regions of the placental methylome. While we also explored whether our findings were enriched for allele-specific gDMRs, only one of the 443 MSDP-associated CpGs was within a candidate maternal gDMR (cg05211790 annotated to *RAI14*), suggesting that gDMRs are not substantially affected by MSDP.

### Phenotype enrichment analyses

We tested for enrichment for phenotypes within the database of Genotypes and Phenotypes (dbGAP) using EnrichR, to understand the types of health outcomes that have been associated with these genes. We again tested the gene lists based on those annotated to our CpGs (*n* = 284 genes) and those mapped to eQTM genes (*n* = 211 genes). The Illumina annotated genes were enriched for cell adhesion molecules (CAM), asthma, body mass index (BMI), blood pressure, and antipsychotic agents; while the gene list based on eQTM mapping did not yield significantly enriched phenotypes (Supplementary Data 15 and 16). The CAM designation in dbGAP, which was the most significantly enriched phenotype (Fisher’s exact *p* value = 6.51E−05), describes a broad array of molecular functions related to cellular mobility and integration, wound healing, and metastasis, which are related to many of the biological functions identified in the above pathway enrichment tests. While the other phenotypes are related to some of the health outcomes that have been associated with prenatal tobacco smoke exposure: asthma, cardiometabolic effects (BMI and blood pressure), and psychiatric effects (antipsychotic agents).

### Proximity to genetic variants linked to birth outcomes

We aimed to understand whether the regions of the placental genome where DNAm is responsive to MSDP are also important in fetal growth regulation. Thus, we explored whether genetic variants that have previously been associated with birth outcomes via genome-wide association studies (GWAS) are within close proximity to our identified CpGs. We examined whether MSDP-associated CpGs were within ±0.5 Mb (1 Mb window) of single-nucleotide polymorphisms (SNPs) that have been associated with BW, birth length (BL), head circumference (HC), and GA^29–34^ (Supplementary Data 8). Of the 330 birth outcome SNPs in autosomal chromosomes, 61 SNPs were within 0.5 Mb of 51 CpGs (Supplementary Data 17), including cg27402634 (*LEKR1*), cg26843110 (*EDC3*), and cg20340720 (*WBP1L*), suggesting that these genomic regions that are responsive to MSDP appear to be involved in growth regulation. We also explored whether our MSDP-associated CpGs may be biased by methylation quantitative trait loci (mQTLs), in which SNPs influence the methylation levels at nearby CpGs. Two studies have examined this question in human placenta, identifying 866 (ref. ^35^) and 4342 (ref. ^36^) placental mQTLs. Our findings did not appear to be biased by genetic variation as only 5 of the 443 MSDP-associated CpGs were previously characterized placental mQTLs.

### DNAm associated with smoking-related birth outcomes

We then performed secondary meta-analyses to examine the relationships between DNAm with GA at birth, preterm birth, BW, BL, and HC *z*-scores. Of the 443 CpGs tested, 142 (32.1%) were related to at least one birth outcome after Bonferroni adjustment (0.05/443). The majority of birth outcome associations were related to GA at birth (121 CpGs) (Supplementary Data 18). Preterm delivery, produced similar associations, although fewer CpGs were statistically significant (Supplementary Data 19). We also found that numerous CpGs were associated with birth size *z*-scores, with the majority of these being associated with BW (25 CpGs; Supplementary Data 20), followed by BL (11 CpGs; Supplementary Data 21) and HC (2 CpGs; Supplementary Data 22). Some of the CpGs associated with GA were also associated with birth size measurements, even although BW, BL, and HC were standardized for GA, suggesting independent associations with both gestational duration and fetal growth (Supplementary Fig. 7). Four CpGs (annotated to *KDM5B, TTC7B, SFRS1*, and *DUSP6*) shared associations with both GA and BW, two (annotated to *TMEM51* and *MYO7A*) with both GA and BL, and one with all three of GA, BW, and BL (annotated to *KIAA1211*). Among the CpGs that were associated with at least one of these birth outcomes, those that tended to have positive associations with BW, BL, HC, or GA were typically hypomethylated with exposure to MSDP, while CpGs that exhibited inverse associations with birth outcomes tended to be hypermethylated with exposure to MSDP.

**Fig. 3:**
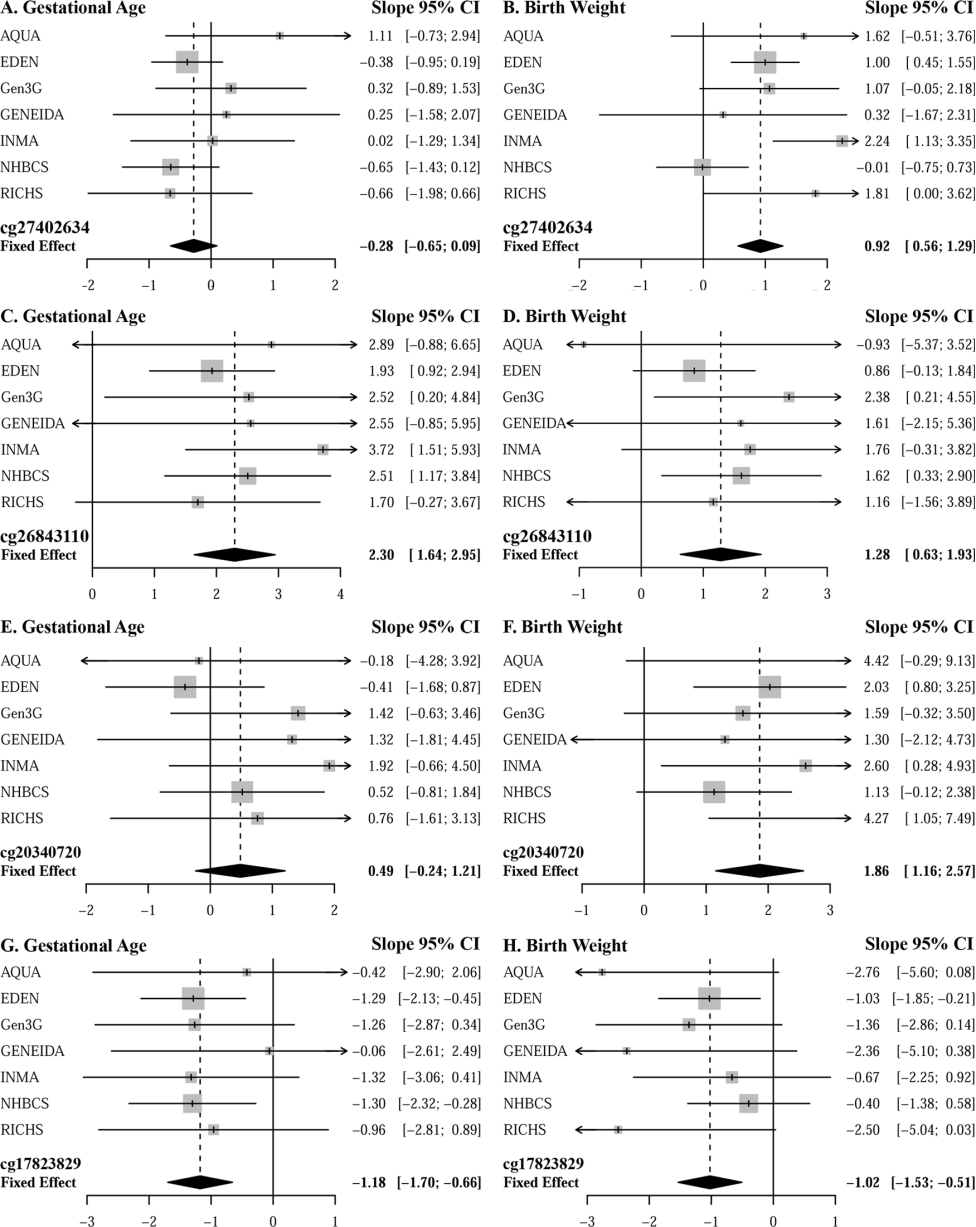
Forest plots of the cohort specific and inverse-variance fixed-effect meta-analysis estimates of association between higher levels of placental DNAm with gestational age at birth and birth weight. Estimated differences in gestational age at birth and birth weight *z*-scores (slope) and 95% confidence intervals (95% CI) associated with increasing levels of DNAm at **A**, **B** cg27402634, **C**, **D** cg26843110, **E**, **F** cg20340720, and **G**, **H** cg17823829. All models were adjusted for maternal age, parity, maternal education, and putative cellular heterogeneity. The slopes and 95% confidence intervals (95% CIs), represent the regression coefficients from linear models, in which gestational age (inverse normal transformed) and birth weight (*z*-scores) were regressed on DNAm at each CpG, while adjusting for confounders.

Among our top 20 CpGs that were associated with any MSDP, 5 were associated with GA at birth and 6 were associated with BW *z*-scores (Table 3). DNAm at cg27402634 (*LEKR1*) and cg20340720 (*WBP1L)*, both located close to BW-SNPs and for which MSDP associated with lower DNAm, were associated with larger BW (*p* value = 6.71E−07 and *p* value = 2.42E−07, respectively). On the other hand, DNAm at cg26843110 (*EDC3;* hypomethylated in response to MDSP and also close to BW-SNPs) and at cg17823829 (*KDM5B;* hypermethylated) were associated with longer and shorter GAs at birth, respectively (*p* value = 5.09E−12 and *p* value = 9.11E−06, via inverse-variance fixed-effect meta-analysis). Forest plots of BW *z-*scores and GA for these four CpGs are shown in Fig. 3.

### Comparison with CpGs associated with MSDP in cord blood

We then assessed whether the DNAm signatures of MSDP in the placenta were consistent with MSDP associations in cord blood previously reported by the PACE consortium^10^. Only four CpGs (annotated to *CYP1A1*, *GNG12*, *RNF122*, and *ZBTB4*) yielded significant associations in both tissues (Table 4). Of note, the CpGs within *CYP1A1* and *RNF122* showed opposite directions of association with MSDP in cord blood and placenta. There was no overall genome-wide correlation (*r*^2^ < 0.1) of the regression coefficients across these two tissues (Supplementary Fig. 8). We also explored whether there was more consistency if we used a relaxed significance threshold. Of the 6073 CpGs that were within a 5% FDR from the cord blood analysis, 115 also yielded associations with FDR < 5% in placenta, 70 (61%) of which had consistent directions of effect between the two tissues (Supplementary Data 23). Among these, one CpG within *AHRR*, cg21161138, exhibited consistent hypomethylation with MSDP across both studies; this was notable since CpGs within the *AHRR* gene have been most consistently identified in studies of MSDP and cord blood DNAm.

**Table 4:** Comparing MSDP-associated CpGs from placenta to those from cord blood.

CpG	Gene	Cord *β*_1_	Cord *p*	Plac._any_ *β*_1_	Plac._any_ *p*	Plac._sust._ *β*_1_	Plac._sust_ *p*	Dir.
cg06397161	*SYNGR1*	−0.008	3.44E−06	0.034	3.25E−14	0.046	6.66E−13	−/+
cg07565956	*ZBTB4*	−0.006	**2.50E−10**	−0.014	6.20E−12	−0.021	1.10E−12	−/−
cg08327744	*RNF122*	−0.010	**1.37E−08**	0.017	2.57E−08	0.022	2.26E−08	−/+
cg14351425	*GRK5*	0.007	1.09E−06	0.027	2.27E−08	0.037	1.21E−08	+/+
cg14541011	*RALGDS*	−0.007	2.99E−07	0.019	2.17E−09	0.022	2.63E−07	−/+
cg16704246	*RBM20*	−0.008	6.21E−07	−0.017	4.03E−11	−0.023	6.17E−10	−/−
cg23160522	*CYP1A1*	0.006	2.33E−06	−0.029	2.60E−07	−0.042	2.48E−08	+/−
cg23680900	*CYP1A1*	0.003	**8.73E−08**	−0.005	1.52E−03	−0.016	6.88E−14	+/−
cg25189904	*GNG12*	−0.024	**1.38E−32**	−0.014	3.80E−08	−0.019	1.64E−09	−/−

We present the meta-analysis results for CpGs among our 443 MSDP-associated CpGs that were also associated with MSDP in cord blood (from a published PACE meta-analysis^10^) with *p* values < 1E−05; those that passed a Bonferroni-corrected threshold in the cord blood study are highlighted in bold.

*β*_1_ coefficient of the association between DNAm and MSDP, which represent the estimated difference in the proportion of DNAm at each CpG when comparing mothers that smoked during pregnancy (MSDP), to those that did not smoke during pregnancy; *p* values are from inverse-variance fixed-effect meta-analysis, *MSDP* maternal smoking during pregnancy, *Dir.* direction of effect first for cord blood, then for placenta, *Plac.* results from placental tissue.

## Discussion

We identified 443 CpG sites with placental methylation levels that were associated with any or sustained MSDP. Differential DNAm was greater for the majority of these CpGs when we stratified to sustained MSDP, and a large proportion of the MSDP-associated CpGs were related to birth outcomes. Those CpGs that were observed to have higher DNAm associated with MSDP, tended to be inversely associated with GA and birth size, while CpGs exhibiting lower DNAm with MSDP tended to be positively associated with GA and birth size.

The most statistically significant association (cg27402634), also identified in prior EWAS of MSDP in placental tissues^15^, yielded dramatically lower DNAm levels in association with MSDP exposure. This effect size is much larger in magnitude (~25% difference for sustained MSDP) compared to what has generally been observed in most exposure-focused EWAS, although within the same range as a CpG site in *AHRR* (cg05575921; 18% difference between exposed and unexposed) from a prior EWAS of current smoking and blood DNAm^37^. In addition, decreased placental DNAm at cg27402634 correlates with increased expression of *LEKR1*, and associates with smaller BW and BL. Thus MSDP-associated hypomethylation at this CpG would be consistent with the well-known effect of maternal smoking, resulting in shorter gestation and smaller birth size.

The functional activities of cg27402634, or corresponding *LEKR1* gene, in human placental tissues are not known. However, GWAS findings provide evidence that genetic variants within this region (3q25.31) might be involved in fetal growth and possibly metabolic programming. For instance, the SNP rs1482852 or its proxies (rs900400; rs13322435) have been associated with fetal growth^38^, adiposity in newborns^39,40^, maternal adiponectin levels, cord blood leptin^40^, and insulin release after an oral glucose challenge^41^. These findings from genetic studies in combination with our current study, implicate that this locus on chromosome 3 (3q25.31) contains active determinants of fetal growth regulation and metabolic activity, and that placental DNAm at cg27402634 is highly responsive to maternal smoking. Future mechanistic work is necessary to investigate whether the placental epigenetic regulation at this locus specifically influences placental functions and/or overall growth and metabolic functions of the developing fetus.

We identified numerous other notable MSDP-associated CpGs, and highlight those CpGs yielding the strongest magnitudes of effect (cg20340720, cg26843110, and cg17823829). MSDP was associated with lower DNAm at cg20340720, located within *WBP1L* (also annotated as *C10orf26*), while lower DNAm at this CpG correlated with lower with BW and BL *z*-scores. Genetic variants nearby to this CpG have been related to BW^34^ and blood pressure^42^. We also observed lower DNAm with MSDP at cg26843110, which is within the body of the *EDC3* gene, and is nearby to SNPs associated with BW (rs3784789, ref. ^34^). Lower DNAm at cg26843110 associated with shorter GA at birth, and decreased expression of *CSK*, which is involved in trophoblast differentiation^43^, as well as blood pressure and aldosterone regulation^44^. Finally, cg17823829 (annotated to *KDM5B*) was hypermethylated with MSDP. Higher DNAm at this CpG correlated with shorter GA at birth and with lower expression of *PPFIA4* gene, which can be induced in response to hypoxia^45^.

Our enrichment analyses identified numerous interrelated pathways that are critical to placental growth and development. The Ahr pathway was significantly enriched among the eQTM-mapped genes, and is well recognized for its roles in responding to environmental exposures and influencing immune activity, particularly among Th17 cells^46^. Multiple pro-inflammatory immune cell pathways, such as Th17 cell differentiation, as well as neutrophil and platelet degranulation were also enriched among these MSDP-associated eQTM genes. Th17 cells can induce inflammation and oxidative stress in the placenta^47^, while neutrophils are involved in the inflammatory cascades that are thought to contribute to preterm birth^48^. Platelets can recognize damaged tissues and coordinate T-cell-mediated inflammatory responses, including Th17 cells^49^. Receptor tyrosine kinase pathways, which were enriched among both the eQTM and Illumina annotated gene sets, describe a broad array of cell surface receptors that can bind to cytokines, hormones, and growth factors, and include the epidermal growth factor receptor (EGFR). The EGFR is highly expressed in placental tissues, while EGF is involved in protecting trophoblasts from hypoxia-induced apoptosis^50^, and perturbed EGF-EGFR signaling has been associated with placental pathologies and growth restriction^51^. In addition, when activated, the EGFR initiates multiple signaling pathways involving MAPK/JAK kinases or STAT TFs^52^, while the *MAPKAPK3*, *JAK1*, and *STAT5A* genes were among those with MSDP-associated CpGs and were involved in numerous pathways identified in our enrichment analyses. Thus, these functional enrichment analyses characterized numerous interrelated pathways that contribute to sensing and responding to environmental stressors, regulating placental inflammatory activity, and influencing growth factor signaling.

We aimed to better understand the overall regulatory landscape of these CpGs by testing for enrichment for regulatory features (TSSs, enhancers, gene bodies, and untranslated regions), PMDs, allele-specific gDMRs, and whether they were enriched for TF binding and phenotypes via EnrichR. These CpGs were enriched in placental enhancers while depleted in PMDs^28^, suggesting that they are located within active regulatory regions. The genes that were annotated to MSDP-associated CpGs were enriched for genes regulated by specific TFs, most notably GATA1, GATA2, and RUNX1. Together with PPARG and TP63, GATA factors are part of the core transcriptional regulatory circuit that guides and maintains proper trophoblast differentiation^53,54^, while angiogenic activity is reduced in placentas lacking *GATA2*^55^. RUNX1 on the other hand, is a driver of haematopoietic development^56^, and while not thoroughly studied in human placenta, there is some evidence that placental inflammation is associated with the upregulation of placental RUNX1 and hematopoiesis^57^. Genes that were annotated to MSDP-associated CpGs have also been linked to human health and disease traits via dbGAP, including a number of conditions related to cardiometabolic health (BMI and blood pressure), which has previously been linked to MSDP^58,59^. This may indicate that MSDP effects placental genes that are involved in energy uptake and expenditure, lipid and glucose metabolism, blood pressure regulation, and inflammation, which are some of the key physiological processes that are disrupted in the pathogenesis of metabolic syndrome^60^. Genes were also enriched for asthma and psychiatric health, which are known to be related to MSDP^61^. Thus, the epigenetic response to MSDP predominantly occurs at CpGs that are in active regions of the placental genome, at genes that are targeted by TFs involved in angiogenesis and hematopoiesis, and within genes that have been linked to cardiometabolic, respiratory, and psychiatric health.

We also aimed to understand whether the regions of the epigenome that associate with MSDP are involved in fetal growth regulation. We found that many of these CpGs, including some of our strongest hits from the meta-analysis, were in similar genomic proximity (within 0.5 Mb) to SNPs that have previously been associated with birth size or GA at birth^29–34^. In addition, our secondary meta-analyses demonstrated that DNAm levels at almost half of our CpGs were also associated with GA and/or BW and BL. Thus, the CpG and gene lists that we have produced in this study are robustly associated with MSDP and with birth outcomes across multiple independent populations, making these compelling candidates for future studies aimed at performing causal mediation analyses. We did not pursue mediation in this study as it has been established that due to measurement error in self-reported smoking and the fact that smoking-related methylation is an excellent biomarker of exposure, mediation can be overestimated^62^. Thus, mediation studies are most suitable for cohorts that collect objective smoking exposure biomarkers, such as cotinine or 4-(methylnitrosamino)-1-(3-pyridyl)-1-butanol (NNAL). Some studies, however, have demonstrated that placental DNAm is an interesting candidate as a possible mediator between MSDP and lower BW^15,63^, and we encourage additional investigation of this possibility (while also considering other birth outcomes), using the CpGs that we identified as candidates and using Mendelian Randomization approaches. It is also possible that MSDP, genetic variation, and placental DNAm in these regions could yield additive or interactive effects on birth outcomes. Prior studies have addressed this question in blood^64,65^, but future research is needed to explore the potential interactive effects between genetic variants and MSDP in relation to DNAm in the placenta or between genetic varaints and MSDP-associated DNAm in relation to birth outcomes. In addition, while our MSDP-associated CpGs are in similar genomic regions (1 Mb windows) with birth outcome SNPs, very few of our CpGs (4 of 443) are known placental mQTLs^35,36^, and thus we do not think that our findings were substantially biased by nearby genetic variation.

We compared our findings to those of a previous PACE meta-analysis of MSDP and cord blood DNAm^10^. The most statistically significant association with MSDP in placenta (cg27402634, *LEKR1*) was not associated with MSDP in the cord blood meta-analysis. Only two CpG sites, annotated to *GNG12* and *ZBTB4*, were differentially methylated at genome-wide threshold of statistical significance in both placenta and cord blood, with the same direction of association in both tissues. While two CpG sites within *CYP1A1* and *RNF122* were genome-wide significant in both meta-analyses, but with different directions of association in cord blood versus placenta. Interestingly, we observed *CYP1A1* to be hypomethylated in placenta with exposure to MSDP, which is consistent with studies of adipose, skin, and lung tissues^66^, but this CpG was hypermethylated in cord blood^10^. Even when using relaxed significance thresholds, such as FDR, only 70 CpGs exhibited consistent effects between these two tissues. One CpG within *AHRR* (cg21161138) did exhibit consistent hypomethylation with MSDP across both tissues at this relaxed FDR significance threshold. These observations suggest that there are unique placenta-specific CpG methylation responses to this exposure. However, as mentioned above, the Ahr pathway, was significantly enriched among our eQTM genes, and the cord blood study did identify some genes involved in pro-inflammatory response and growth factor signaling. Thus, while there was little overlap in specific MSDP-associated CpGs and genes between these two tissues, there was some overlap in the overall biological processes.

The above findings should be interpreted within the context of this study’s limitations. MSDP was self-reported and subject to misclassification, although differential misclassification most likely would have biased our findings toward the null. We found that sustained MSDP produced larger magnitudes of association, as has been previously found in studies of blood DNAm^67^, but we did not explicitly compare those mothers that smoked throughout pregnancy to those that quit during early pregnancy, nor did we assess dose–response patterns (i.e., number of cigarettes or biomarker concentrations), both of which should be the focus of future investigations. Our study predominantly consisted of samples from mother–infant pairs of European ancestry, and thus additional studies involving diverse ancestries and ethnic backgrounds are needed, in order to improve the generalizability of these findings. The observed associations between DNAm levels and reproductive outcomes could be due to reverse causation, which is one of the reasons we did not pursue formal mediation analyses. In addition, placenta is a heterogeneous tissue with multiple different cell types^68^ that serve different functions and thus have different epigenetic states^69^. To correct for this, we estimated and adjusted for variability using a data-driven approach, RefFreeCellMix^22^, since no references for placental cell-type methylomes was available at the time. In addition, it is possible that cohort-specific sampling protocols or other cohort-specific differences in data generation could have resulted in placental samples from some cohorts having greater heterogeneity than others. Residual confounding from cellular heterogeneity or other unmeasured confounders may have contributed to the observed inflation in our original meta-analyses, thus we implemented BACON^23^ and only present those findings that were statistically significant after applying Bonferroni correction for multiple testing after inflation and bias correction with BACON.

Despite these limitations, our study had numerous strengths, including a large sample size across seven independent studies, harmonized definitions of exposure variables and covariates, and standardized protocols for quality control, pre-processing and analyses of DNAm data. We performed secondary analyses involving mRNA expression, functional and phenotype enrichment, overlap with GWAS hits for reproductive outcomes, and meta-analyses of DNAm variation with birth outcomes to provide biological and health-related interpretations of our findings. Overall, we identified a DNAm signature of MSDP in the placenta that shows substantial differences from that observed in cord blood, most notably cg27402634 which is intergenic between *LINC00886* and *LEKR1*, where placentas that were exposed had ~25% lower DNAm than those that were not exposed. The MSDP-associated CpGs are within active regions of the placental epigenome, and the genes that are associated with them are involved in responding to environmental stressors, regulating inflammatory activity, signaling through growth factors, and have previously been related to cardiometabolic outcomes. In addition, many of these CpGs were within similar genomic proximity to birth outcome SNPs, and we demonstrated that DNAm was also associated with GA at birth or birth size. These CpGs, genes, and biological pathways provide compelling candidates for follow-up studies aimed at testing causal mediation or at elucidating mechanisms.

## Methods

### Participating cohorts

Cohorts that are members of the PACE consortium were identified for participation in the current study, if they had existing DNAm data quantified from placental tissue via the Illumina Infinium HumanMethylation450 BeadChip, and if they had obtained information on self-reported smoking during pregnancy. The seven cohorts that contributed to the meta-analysis of any MSDP included AQUA^16^, EDEN^17^, Gen3G^18^, GENEIDA, INMA^19^, NHBCS^20^, and RICHS^21^. EDEN, GENEIDA, and INMA also contributed to the sustained MSDP stratified analyses. RICHS contributed RNA-seq data for analyses with mRNA expression. All cohorts acquired ethics approval and informed consent from participants prior to data collection through local ethics committees. Exclusion criteria for this study were non-singleton births, preeclampsia, and DNAm not assessed in the fetal side of the placenta. All participants in the study were of European ancestry, except 1.85% of EDEN mothers. Detailed methods for each cohort are provided in the Supplementary Material (Supplementary Methods File).

### Tobacco smoking definitions

Any MSDP was defined as mothers reporting smoking cigarettes at any time during pregnancy. Sustained MSDP was defined as mothers reporting smoking cigarettes in the first and third trimester of pregnancy. For both exposure variables, the comparison group was defined as the mothers that reported no smoking during any of the pregnancy.

### DNAm data quality control and normalization

All DNAm data processing and analyses were conducted in R, with the exception of the meta-analyses, which were performed with METAL. Placental DNAm from the fetal side was assessed with the Infinium HumanMethylation450 array (Illumina, San Diego, CA USA). See Supplementary Methods file for extra details on placenta collection, DNA extraction, and DNAm acquisition in each cohort. Quality control of DNAm was standardized across all cohorts. Low-quality samples were filtered out and probes with detection *p* values > 0.01 were excluded. Beta values were normalized via functional normalization^70^, and beta-mixture quantile normalization^71^ was applied to correct for the probe type bias. Cohorts searched their data for batch effects and applied ComBat when applicable; all but one cohort (GENEIDA) identified batch effects and used ComBat to remove this source of variation (Supplementary Data 2). Probes that hybridize to the X/Y chromosomes, cross-hybridizing probes, and probes with SNPs at the CpG site, extension site, or within 10 bp of the extension site with an average minor allele frequency > 0.01 were filtered out^72^. Overall, 418,658 probes and 415,396 were available for modeling any MSDP and sustained MSDP, respectively. Finally, DNAm extreme outliers (<25th percentile − 3 × IQR or >75th percentile + 3 × IQR across all the samples) were trimmed.

### Estimates of putative cellular heterogeneity

Placental putative cellular heterogeneity was estimated from DNAm data using a reference-free cell-mixture decomposition method (RefFreeCellMix)^73^. The number of components varied between cohorts ranging from two to five components, which could be due to different sampling protocols, resulting in differential heterogeneity across cohort, or since this approach is data driven these components could be capturing other major sources of variation in the array data, such as residual technical artifacts. Models for differential DNAm were corrected for the number of surrogate variables minus one to reduce multi-collinearity.

### Genome-wide differential DNAm analyses

Within each cohort, robust linear regression from the MASS package^74^ in R were used to account for potential heteroskedasticity, while testing the associations between normalized DNAm beta values at each CpG with any MSDP and sustained MSDP. Models were adjusted for maternal age, parity, maternal education, and cohort-specific variables first unadjusted for putative cellular heterogeneity then adjusted for RefFreeCellMix estimates of putative cellular heterogeneity. Due to the inflation and potential residual or unmeasured confounding, we applied BACON to the cohort-specific results before performing meta-analyses. BACON was specifically developed for EWAS and estimates an empirical null distribution to correct for residual bias and inflation^23^. We performed inverse-variance weighted fixed-effect meta-analyses using METAL^75^. The meta-analysis was performed independently by two groups to ensure consistent results. Both groups detected some inconsistent errors within some cohort-specific results, and the cohorts were contacted to redo the analysis. After these were addressed, results between independent groups were completely consistent. A third group then used an independent R script to perform meta-analysis with the metafor package, and again successfully reproduced identical results. CpGs not retained in at least two cohorts were filtered out. We used the Bonferroni adjustment to control for multiple testing. To examine whether differential methylation associated with sustained smoking yielded stronger magnitudes of effect relative to models of any smoking, we calculated the percent change in the coefficients between the two models (|*β*_sustained_| −  |*β*_any_|)/|*β*_any_| × 100. Secondary analyses were only performed on CpGs that passed a Bonferroni-corrected threshold for associations with any or sustained MSDP in models that were adjusted for RefFreeCellMix and corrected for residual bias via BACON.

### Expression quantitative trait methylation

We performed eQTM^76^ analyses in the RICHS cohort. Transcription was measured via RNA-seq on 194 placentas. The details of sample collection, assay, and QC for the RNA-seq data are presented in detail elsewhere^77^, and summarized in the Supplementary Material (Supplementary Methods File). In this dataset, we identified 2567 unique transcripts annotated to an Ensembl ID (GrCh37/hg19) and with a TSS within 250 kb upstream or downstream of 423 out of the 445 candidate CpGs. The association between DNAm and expression levels was assessed via 3507 linear regression models, using the MEAL package^78^ in R; these models were adjusted for RNA-seq batch, DNAm batch, the RICHS selection factor (small, appropriate, or large for GA), and self-reported maternal ancestry. In addition, principal components analyses revealed that the first four components explained 40% of the total variation in the expression data, and they were incorporated as covariates in the model. We annotate CpGs with the eQTM genes that yielded *p* values < 0.05, while statistically significant eQTMs were determined at a Bonferroni-corrected threshold (*α* = 1.43E−05).

### CpG site annotation

We annotated CpGs to genes and CpG islands with notations from the Illumina HumanMethylation450 K annotation file, and with several regulatory features using publicly available data: placental 15-chromatin states^79^ released from the ROADMAP Epigenomics Mapping Consortium^27^ (ChromHMM v1.10), placental PMDs^28^, and placental gDMRs^26^.

### Enrichment analyses

Functional enrichment analyses were performed at the gene level via ConsensusPathDB^80^ using KEGG, Reactome, Wikipathways, and Biocarta as reference gene sets and restricting enrichment to include at least four genes from our gene lists. ConsensusPathDB performs a hypergeometric test and corrects multiple testing with FDR. Enrichment for TFs and for phenotypes were assessed at the gene level with EnrichR, using ENCODE and ChEA consensus TFs from ChIP-X database, and dbGaP database, respectively. EnrichR results were ranked using the combined score (*p* value computed using Fisher exact test combined with the *z-*score of the deviation from the expected rank)^25^. Enrichment for regulatory features was assessed with the hypergeometric test, and *p* values were Bonferroni corrected for 15 (placental chromatin 15 states) and 6 (relation to CpG island) tests, respectively.

### Overlap of MSDP-sensitive CpG sites and birth outcome SNPs

Overlapping genomic regions between MSDP-associated CpGs in placenta with previously identified BW, BL, HC, and GA SNPs from the largest GWAS to date^29–34^ was assessed using the GenomicRanges package in R^81^. We identified which CpGs were located within 1 Mb windows (±0.5 Mb) surrounding each of the 324 autosomal SNPs, which correspond to 280 potential unique loci. Unique loci were defined based on the criteria in Warrington et al.^34^, and linkage disequilibrium in Europeans (*r*^2^ > 0.1 in <2 Mb).

### Association between DNAm and birth outcomes

Within each cohort, robust linear regression models were utilized to test the association between normalized DNAm beta values at each CpG as the independent variable and GA at birth (inverse normal transformation of sex residuals), BW *z*-scores, BL *z*-scores, and HC *z*-scores as the dependent variables. Logistic regression was used to examine the relationships between DNAm and preterm birth (defined as <37 weeks of gestation). Birth size *z*-scores were calculated using international references from the INTERGROWTH-21st Project^82^ and standardized by both GA and newborn sex. Models were adjusted for maternal age, parity, maternal education, cohort-specific variables (see Supplementary Methods), and putative cellular heterogeneity. Inverse-variance weighted fixed-effect meta-analyses^75^ were again used to estimate pooled associations. Multiple testing was controlled with the Bonferroni adjustment (*α* = 0.05/443).

### Comparison with CpGs associated with MSDP in cord blood

We examined the consistency between MSDP-sensitive CpGs in placenta and in cord blood^10^. First, we checked whether MSDP-sensitive CpGs identified in placenta were also reported in cord blood with the same direction of the effect. Then, we compared the coefficients from the models for sustained MSDP in cord blood, unadjusted for cellular heterogeneity, to results for both any and sustained MSDP in placenta, adjusted for cellular heterogeneity, using Pearson correlation coefficients.

### Reporting summary

Further information on research design is available in the Nature Research Reporting Summary linked to this article.

## Supplementary information

Supplementary Information

Description of Additional Supplementary Files

Supplementary Data 1-23

Reporting Summary

## Data Availability

All relevant data supporting the key findings of this study are available within the article and its Supplementary Information files, or from the corresponding author upon reasonable request. A reporting summary with additional information is also included. The complete summary statistics from these meta-analyses are available at FigShare (any MSDP [10.6084/m9.figshare.12198471]) and (sustained MSDP [10.6084/m9.figshare.12198504]). The raw fastq files for placental RNA-seq measurement are available in the NCBI database for Genotypes and Phenotypes (dbGaP) under accession number phs001586.v1.p1.

## Code availability

All of the software packages and programs used to conduct these analyses are freely available, and can be found via the citations in the manuscript and in the links provided in the “Reporting summary”. The code used for these analyses are available upon request to the corresponding authors.
