# Supplementary material for: Placental DNA methylation signatures of maternal smoking during pregnancy and potential impacts on fetal growth
Source: Nat Commun. 2021 Aug 24;12:5095. doi: 10.1038/s41467-021-24558-y (PMC8384884; doi:10.1038/s41467-021-24558-y)
Supplement: Supplementary file 2 — Description of Additional Supplementary Files [file 41467_2021_24558_MOESM2_ESM.pdf]

Description of Additional Supplementary Files

File Name: Supplementary Data 1
Description: Distribution of maternal smoking during pregnancy, demographic vriables, birth outcomes, and covariates, by cohort. MSDP = Maternal Smoking During Pregnany; SD = Standard Deviation.

File Name: Supplementary Data 2
Description: Proportion of maternal smokers that were included in each model, GIF and number of significant CpGs for each model, for the meta-analyses and for individual cohorts; cohort-specific estimates of bias and inflation via BACON, as well as the number of CpGs within a 5% FDR and Bonferroni-corrected threshold have been added. MSDP = Maternal Smoking During Preganncy; FDR = False Discovery Rate; GIF - Genomic Inflation Factor.

File Name: Supplementary Data 3
Description: Meta-analysis results for the association between any MSDP and placental DNAm, while controlling for maternal age, parity, and maternal education, but not adjusted for putative cellular heterogeneity; included data from AQUA, EDEN, Gen3G, GENEIDA, INMA, NHBCS, & RICHS. HetIsq = Heterogeneity I2 Value; HetPVal = P-value for Heterogeneity Test; Chr = Chromosome; Pos = Position (hg19); UCSC_RefGene_Name = Gene Annotatsions from the Illumina Annotation file; UCSC_RefGene_Group = Within Gene location from the Illumina Annotation file.

File Name: Supplementary Data 4
Description: Meta-analysis results for the association between sustained MSDP and placental DNAm, while controlling for maternal age, parity, and maternal education, but not adjusted for putative cellular heterogeneity; included data from EDEN, GENEIDA & INMA. HetIsq = Heterogeneity I2 Value; HetPVal = P-value for Heterogeneity Test; Chr = Chromosome; Pos = Position (hg19); UCSC_RefGene_Name = Gene Annotatsions from the Illumina Annotation file; UCSC_RefGene_Group = Within Gene location from the Illumina Annotation file.

File Name: Supplementary Data 5
Description: Meta-analysis results for the association between any MSDP and placental DNAm, while controlling for maternal age, parity, maternal education, and putative cellular heterogeneity; included data from AQUA, EDEN, Gen3G, GENEIDA, INMA, NHBCS, & RICHS. HetIsq = Heterogeneity I2 Value; HetPVal = P-value for Heterogeneity Test; Chr = Chromosome; Pos = Position (hg19); UCSC_RefGene_Name = Gene Annotatsions from the Illumina Annotation file; UCSC_RefGene_Group = Within Gene location from the Illumina Annotation file.

File Name: Supplementary Data 6
Description: Meta-analysis results for the association between sustained MSDP and placental DNAm, while controlling for maternal age, parity, maternal education, and putative cellular heterogeneity; included data from EDEN, GENEIDA & INMA. HetIsq = Heterogeneity I2 Value; HetPVal = P-value for Heterogeneity Test; Chr = Chromosome; Pos = Position (hg19); UCSC_RefGene_Name = Gene Annotatsions from the Illumina Annotation file; UCSC_RefGene_Group = Within Gene location from the Illumina Annotation file.

File Name: Supplementary Data 7
Description: Among the CpGs that were significantly associated with MSDP after Bonferroni correction, the proportion of CpGs with heterogeneity p-values < 0.01.

File Name: Supplementary Data 8
Description: Meta-analysis results for the association between any- or sustained- MSDP and placental DNAm, while controlling for maternal age, parity, maternal education, putative cellular heterogeneity, and residual bias (via BACON); models for any MSDP included data from AQUA, EDEN, Gen3G, GENEIDA, INMA, NHBCS, & RICHS, while models for sustained MSDP included data from EDEN, GENEIDA & INMA. HetIsq = Heterogeneity I2 Value; HetPVal = P-value for Heterogeneity Test; Chr = Chromosome; Pos = Position (hg19); UCSC_RefGene_Name = Gene Annotatsions from the Illumina Annotation file; UCSC_RefGene_Group = Within Gene location from the Illumina Annotation file; PMD = Partially Methylated Domain; SNP = Single Nucleotide Polymorphism.

File Name: Supplementary Data 9
Description: Results from the expression quantitative trait methylation (eQTM) analysis that yielded raw p-values < 0.05; Ensembl ID for the transcript included in the analysis; GeneStrand = the strand that the eQTM gene is located; GeneType = Ensembl categorization for the transcript type; TSS = genomic location fot the eQTM gene's transcription start site; Chr = Chromosome that the CpG is on; Pos = Position (hg19) of the CpG; UCSC_RefGene_Name = CpG Gene Annotatsions from the Illumina Annotation file; UCSC_RefGene_Group = gene location for the CpG from the Illumina Annotation file.

File Name: Supplementary Data 10
Description: Lists of the eQTM genes and Illumina Annotated genes that were used for functional enrichment analyses; the gene annotations that needed to be manually recoded in order to have an identifiable HGNC gene name are listed in the last column.

File Name: Supplementary Data 11
Description: Pathway enrichment was performed with Consensus Path Database (CPDB) utilizing pathways from KEGG, Biocarta, Reactome, and Wikipathways while requiring a minimum of four overlapping genes between our gene set and a pathway, and adjusting for multiple testing with the false dicovery rate (FDR); This gene set include the 284 genes annotated to the 443 MSDP-associated CpGs; gene annotations were obtained from the Illumina annotation file and when a CpG was annotated to more than one gene, the first gene within the set of genes was used so that each CpG only had a single gene-annotation.

File Name: Supplementary Data 12
Description: Pathway enrichment was performed with CPDB utilizing pathways from KEGG, Biocarta, Reactome, and Wikipathways while requiring a minimum of four overlapping genes between our gene set and a pathway, and adjusting for multiple testing with the FDR; this gene set includes the 211 genes whose expression was associated with the DNA methylation levels of the 443 MSDP-associated CpGs (p-value < 0.05; eQTM genes that were significant after Bonferroni-correction are highlighted in bold text); when a CpG was associated with more than one gene, the gene with the smallest p-value was used for enrichment analyses so that each CpG only had a single gene-annotation.

File Name: Supplementary Data 13
Description: Transcription factor (TF) enrichment was performed with EnrichR utilizing Chip-seq data from ENCODE and ChEA and adjusting for multiple testing with the false dicovery rate (FDR); this gene set included the 284 genes that are annotated to the 443 MSDP-associated CpGs; gene annotations were obtained from the Illumina annotation file and when a CpG was annotated to more than one gene, the first gene within the set of genes was used so that each CpG only had a single gene-annotation.

File Name: Supplementary Data 14
Description: TF enrichment was performed with EnrichR utilizing Chip-seq data from ENCODE and ChEA and adjusting for multiple testing with the false dicovery rate (FDR); this gene set includes the 211 genes whose expression was associated with the DNA methylation levels of the 443 MSDP-associated CpGs (p-value < 0.05; eQTM genes that were significant after Bonferroni-correction are highlighted in bold text); when a CpG was associated with more than one gene, the gene with the smallest p-value was used for enrichment analyses so that each CpG only had a single gene-annotation.

File Name: Supplementary Data 15
Description: Phenotype enrichment was performed with EnrichR utilizing dbGAP and adjusting for multiple testing with the false dicovery rate (FDR); this gene set included the 284 genes that are annotated to the 443 MSDP-associated CpGs; gene annotations were obtained from the Illumina annotation file and when a CpG was annotated to more than one gene, the first gene within the set of genes was used so that each CpG only had a single gene-annotation.

File Name: Supplementary Data 16
Description: Phenotype enrichment was performed with EnrichR utilizing dbGAP and adjusting for multiple testing with the false dicovery rate (FDR); this gene set includes the 211 genes whose expression was associated with the DNA methylation levels of the 443 MSDP-associated CpGs (p-value < 0.05; eQTM genes that were significant after Bonferroni-correction are highlighted in bold text); when a CpG was associated with more than one gene, the gene with the smallest p-value was used for enrichment analyses so that each CpG only had a single gene-annotation.

File Name: Supplementary Data 17
Description: Maternal and fetal SNPs that have been associated with birth size and gestational age in prior studies, annotated with MSDP-associated CpGs in placenta that are within 0.5 Mb of their genomic locations; SNP = single nucleotide polymorphism, Chr = chromosome, Pos = genomic location.

File Name: Supplementary Data 18
Description: Meta-analysis results for the association between placental DNA methylation and gestational age (inverse normal transformation of sex residuals, in days) adjusted for maternal age, parity, and maternal education, and putative cellular heterogeneity; included data from AQUA, EDEN, Gen3G, GENEIDA, INMA, NHBCS & RICHS. Chr = Chromosome; Pos = Positiong (hg19); UCSC Gene Name = Gene Annotatsions from the Illumina Annotation file; 95% CI = confidence interval for the HetIsq = Heterogeneity I2 Value; Het. P-value = P-value for Heterogeneity Test.

File Name: Supplementary Data 19
Description: Meta-analysis results for the association between placental DNA methylation and preterm delivery (< 37 weeks gestation) adjusted for maternal age, parity, and maternal education, and putative cellular heterogeneity; included data from EDEN & NHBCS. Chr = Chromosome; Pos = Positiong (hg19); UCSC Gene Name = Gene Annotatsions from the Illumina Annotation file; 95% CI = confidence interval for the HetIsq = Heterogeneity I2 Value; Het. P-value = P-value for Heterogeneity Test.

File Name: Supplementary Data 20
Description: Meta-analysis results for the association between placental DNA methylation and birth weight z-score (standardized by sex and gestational age via international reference), adjusted for maternal age, parity, and maternal education, and putative cellular heterogeneity; included data from

AQUA, EDEN, Gen3G, GENEIDA, INMA, NHBCS & RICHS. Chr = Chromosome; Pos = Positiong (hg19); UCSC Gene Name = Gene Annotatsions from the Illumina Annotation file; 95% CI = confidence interval for the HetIsq = Heterogeneity I2 Value; Het. P-value = P-value for Heterogeneity Test.

File Name: Supplementary Data 21
Description: Meta-analysis results for the association between placental DNA methylation and birth length z-score (standardized by sex and gestational age via international reference), adjusted for maternal age, parity, and maternal education, and putative cellular heterogeneity; included data from EDEN, Gen3G, GENEIDA, INMA, NHBCS & RICHS. Chr = Chromosome; Pos = Positiong (hg19); UCSC Gene Name = Gene Annotatsions from the Illumina Annotation file; 95% CI = confidence interval for the HetIsq = Heterogeneity I2 Value; Het. P-value = P-value for Heterogeneity Test.

File Name: Supplementary Data 22
Description: Meta-analysis results for the association between placental DNA methylation and head circumference z-score (standardized by sex and gestational age), adjusted for maternal age, parity, and maternal education, and putative cellular heterogeneity; included data from EDEN, Gen3G, GENEIDA, INMA, NHBCS & RICHS. Chr = Chromosome; Pos = Positiong (hg19); UCSC Gene Name = Gene Annotatsions from the Illumina Annotation file; 95% CI = confidence interval for the HetIsq = Heterogeneity I2 Value; Het. P-value = P-value for Heterogeneity Test.

File Name: Supplementary Data 23
Description: CpGs that were associated with MSDP in the prior cord blood analysis and associated with any MSDP and sustained MSDP in placenta at a more relaxed significance threshold (FDR < 5%), with the same direction of effect for all models across all tissues; Chr. = chromosome, Pos. = genomic position, FDR = False Discovery Rate.
