# Supplementary material for: Placental DNA methylation signatures of maternal smoking during pregnancy and potential impacts on fetal growth
Source: Nat Commun. 2021 Aug 24;12:5095. doi: 10.1038/s41467-021-24558-y (PMC8384884; doi:10.1038/s41467-021-24558-y)
Supplement: Supplementary file 4 — Reporting Summary [file 41467_2021_24558_MOESM4_ESM.pdf]

nature research

Corresponding author(s): Todd M. Everson & Mariona Bustamante

Last updated by author(s): Jun 4, 2021

Reporting Summary

Nature Research wishes to improve the reproducibility of the work that we publish. This form provides structure for consistency and transparency in reporting. For further information on Nature Research policies, see Authors & Referees and the Editorial Policy Checklist .

Statistics

For all statistical analyses, confirm that the following items are present in the figure legend, table legend, main text, or Methods section.

n/a	Confirmed	
☐	☒	The exact sample size (*n*) for each experimental group/condition, given as a discrete number and unit of measurement
☒	☐	A statement on whether measurements were taken from distinct samples or whether the same sample was measured repeatedly
☐	☒	The statistical test(s) used AND whether they are one- or two-sided<br>*Only common tests should be described solely by name; describe more complex techniques in the Methods section.*
☐	☒	A description of all covariates tested
☐	☒	A description of any assumptions or corrections, such as tests of normality and adjustment for multiple comparisons
☐	☒	A full description of the statistical parameters including central tendency (e.g. means) or other basic estimates (e.g. regression coefficient) AND variation (e.g. standard deviation) or associated estimates of uncertainty (e.g. confidence intervals)
☐	☒	For null hypothesis testing, the test statistic (e.g. *F*, *t*, *r*) with confidence intervals, effect sizes, degrees of freedom and *P* value noted<br>*Give P values as exact values whenever suitable.*
☒	☐	For Bayesian analysis, information on the choice of priors and Markov chain Monte Carlo settings
☒	☐	For hierarchical and complex designs, identification of the appropriate level for tests and full reporting of outcomes
☐	☒	Estimates of effect sizes (e.g. Cohen's *d*, Pearson's *r*), indicating how they were calculated

Our web collection on statistics for biologists contains articles on many of the points above.

Software and code

Policy information about availability of computer code

Data collection	The Illumina® Infinium HumanMethylation450 BeadChip was used for measuring DNA methylation.
Data analysis	All software that were used for statistical analyses are freely available. R (V.3.6.1 and later; https://www.r-project.org/); METAL (V.2011-03-25, https://genome.sph.umich.edu/wiki/METAL_Documentation); RefFreeEWAS package (V2.2, https://cran.r-project.org/web/packages/RefFreeEWAS/index.html); MASS package (V7.3, https://cran.r-project.org/web/packages/MASS/index.html), BACON package (V1.2, https://bioconductor.org/packages/release/bioc/html/bacon.html); metafor package (V3.0, https://cran.r-project.org/web/packages/metafor/index.html); GenomicRanges package (V1.4, https://bioconductor.org/packages/release/bioc/html/GenomicRanges.html); ConsensusPathDB (V33, http://cpdb.molgen.mpg.de/); EnrichR (V3.0, https://maayanlab.cloud/Enrichr/). The code used for these analyses and other data are available from the authors upon reasonable request to the corresponding authors.

For manuscripts utilizing custom algorithms or software that are central to the research but not yet described in published literature, software must be made available to editors/reviewers. We strongly encourage code deposition in a community repository (e.g. GitHub). See the Nature Research guidelines for submitting code & software for further information.

Data

Policy information about availability of data

All manuscripts must include a data availability statement. This statement should provide the following information, where applicable:

- Accession codes, unique identifiers, or web links for publicly available datasets
- A list of figures that have associated raw data
- A description of any restrictions on data availability

All relevant data supporting the key findings of this study are available within the article and its Supplementary Information files. The complete summary statistics from these meta-analyses are available at doi: 10.6084/m9.figshare.12198471 (Any MSDP Results) and doi: 10.6084/m9.figshare.12198504 (Sustained MSDP Results). Publicly available databases were used for annotation and to perform enrichment analyses for this project, including ChromHMM (V1.10, http://compbio.mit.edu/ChromHMM/), ConsensusPathDB (http://consensuspathdb.org/) which draws information from KEGG, Reactome, Wikipathways, and Biocarta

databases, and EnrichR (https://maayanlab.cloud/Enrichr/) which draws information from ENCODE and ChEA consensus TFs from ChIP-X and dbGaP databases.

Field-specific reporting

Please select the one below that is the best fit for your research. If you are not sure, read the appropriate sections before making your selection.

[x] Life sciences　　[ ] Behavioural & social sciences　　[ ] Ecological, evolutionary & environmental sciences

For a reference copy of the document with all sections, see nature.com/documents/nr-reporting-summary-flat.pdf

Life sciences study design

All studies must disclose on these points even when the disclosure is negative.

Sample size

We performed EWAS meta-analyses of any smoking during pregnancy (N=1,700, 344 exposed) and sustained smoking throughout pregnancy (N=795, 163 exposed). The only a priori selection criteria for this study was availability of the data required for these analyses (maternal smoking during pregnancy, DNA methylation from placental tissue, and confounders). This is currently the largest epigenome-wide association studies (EWAS) of maternal smoking during pregnancy and placental DNA methylation.

Data exclusions

We had pre-established exclusion criteria to ensure comparability of placental DNA methylation data across cohorts that were included in the meta-analyses, including (1) non-singleton births, (2) pre-eclampsia, and (3) DNAm not assessed in the fetal side of the placenta. For the methylation array data, we excluded probes that suffer from different sources of measurement error, such as those with detection p-values > 0.01, probes that hybridized to the X/Y chromosomes, cross-hybridizing probes and probes with SNPs at the CpG site, extension site, or within 10 bp of the extension site with an average minor allele frequency > 0.01.

Replication

We included all available studies in the meta-analyses to maximize power for discovery; replication was not performed.

Randomization

This was a meta-analysis of observational data, so no randomization was performed.

Blinding

This was a meta-analysis of observational data, so no blinding was performed.

Reporting for specific materials, systems and methods

We require information from authors about some types of materials, experimental systems and methods used in many studies. Here, indicate whether each material, system or method listed is relevant to your study. If you are not sure if a list item applies to your research, read the appropriate section before selecting a response.

Materials & experimental systems

n/a	Involved in the study	
[x]	[ ]	Antibodies
[x]	[ ]	Eukaryotic cell lines
[x]	[ ]	Palaeontology
[x]	[ ]	Animals and other organisms
[ ]	[x]	Human research participants
[x]	[ ]	Clinical data

Methods

n/a	Involved in the study	
[x]	[ ]	ChIP-seq
[x]	[ ]	Flow cytometry
[x]	[ ]	MRI-based neuroimaging

Human research participants

Policy information about studies involving human research participants

Population characteristics

Seven independent cohorts participated in this study, providing data for 1,700 mothers, newborns, and placentas, primarily of Caucasian ancestry with approximately equal proportions of male and female newborns. Detailed reporting of demographics, and other cohort characteristics are provided in Supplementary Data 1.

Recruitment

The seven participating cohorts provide specific details for each cohort and any potential cohort-specific selection factors within the Supplemental Methods file. For this meta-analysis, all cohorts that participate in the PACE consortium that had existing placental DNA methylation data, at least 10 mothers that reported smoking during pregnancy, and that were willing to provide data were included.

Ethics oversight

All studies obtained ethical approval from local committees, and statements about these approvals are included in the Supplementary Methods.

Note that full information on the approval of the study protocol must also be provided in the manuscript.
